# Neuroinflammation as an etiological trigger for depression comorbid with inflammatory bowel disease

**DOI:** 10.1186/s12974-021-02354-1

**Published:** 2022-01-04

**Authors:** Colin F. Craig, Rhiannon T. Filippone, Rhian Stavely, Joel C. Bornstein, Vasso Apostolopoulos, Kulmira Nurgali

**Affiliations:** 1 Institute for Heath and Sport, Victoria University, Western Centre for Health, Research and Education, Sunshine Hospital, Melbourne, VIC Australia; 2 Department of Pediatric Surgery, Pediatric Surgery Research Laboratories, Massachusetts General Hospital, Harvard Medical School, Boston, MA 02114 USA; 3 Department of Anatomy and Physiology, The University of Melbourne, Melbourne, Australia; 4 Immunology Program, Australian Institute of Musculoskeletal Science (AIMSS), Melbourne, VIC Australia; 5 Department of Medicine Western Health, Faculty of Medicine, Dentistry and Health Sciences, The University of Melbourne, Melbourne, VIC Australia; 6 Regenerative Medicine and Stem Cells Program, Australian Institute of Musculoskeletal Science (AIMSS), Melbourne, VIC Australia; 7 Institute for Health and Sport, Victoria University, Level 4 Research Labs, Western Centre for Health Research and Education, Sunshine Hospital, 176 Furlong Road, St Albans, VIC 3021 Australia

**Keywords:** Inflammatory bowel disease, Depression, Neuroinflammation, Gut-brain axis

## Abstract

Patients with inflammatory bowel disease (IBD) suffer from depression at higher rates than the general population. An etiological trigger of depressive symptoms is theorised to be inflammation within the central nervous system. It is believed that heightened intestinal inflammation and dysfunction of the enteric nervous system (ENS) contribute to impaired intestinal permeability, which facilitates the translocation of intestinal enterotoxins into the blood circulation. Consequently, these may compromise the immunological and physiological functioning of distant non-intestinal tissues such as the brain. In vivo models of colitis provide evidence of increased blood–brain barrier permeability and enhanced central nervous system (CNS) immune activity triggered by intestinal enterotoxins and blood-borne inflammatory mediators. Understanding the immunological, physiological, and structural changes associated with IBD and neuroinflammation may aid in the development of more tailored and suitable pharmaceutical treatment for IBD-associated depression.

## Introduction

Inflammatory bowel disease (IBD) is believed to affect up to 7 million people globally with the incidence rising in many western countries [1]. Patients diagnosed with IBD have higher rates of depression and anxiety compared to the general population [2]. Depression is often associates with poorer compliance to treatment regimens and increases the risk of morbidity and mortality of individuals with a chronic medical condition [3, 4]. The gut-brain axis is believed to play a significant role in pathogeneses and/or relapse of IBD symptoms [5]. This review aims to reveal the pathophysiological alterations in the gut and brain in IBD patients and animal models of colitis. It may provide an insight into neurobiological mechanisms, which could be targeted to relieve depression in IBD patients. Better-suited pharmacological approaches to IBD patients with depression will help to relieve the immense psychological burden of this debilitating chronic disease and potentially help to correct the gut-brain axis to prevent the recurrence of intestinal inflammation. Moreover, underlying mechanisms of depression comorbid with IBD may be highly translatable to other diseases such as rheumatoid arthritis, obstructive pulmonary disease, and diabetes, which demonstrate higher rates of depression compared to the general population [6,7,8].

## Background

Inflammatory bowel disease (IBD) is an idiopathic condition that manifests as chronic inflammation within the gastrointestinal (GI) tract and affects approximately 7 million people worldwide [1]. The two major forms of IBD are ulcerative colitis (UC) and Crohn’s disease (CD). UC is characterized by chronic inflammation leading to ulceration which affects primarily the colon and is restricted to the intestinal mucosa layer [9]. On the contrary, CD appears as transmural inflammatory lesions that present anywhere within the GI tract from the oropharynx to perianal areas [9]. Clinical symptoms including abdominal pain, hypersensitivity, diarrhea, blood and mucus in the stools, fatigue, and weight loss are similar between both pathologies of IBD [9]. Although the etiology of IBD remains largely obscure, it has been postulated that elements of an individual’s genetics, environmental exposures, microbiota dysbiosis, and a dysregulated immune response may attribute to IBD pathogenesis [9, 10]. It has been established that structural and functional abnormalities of the enteric nervous system (ENS), the intrinsic innervation of the GI tract, are associated with recurrence of symptoms and disease severity of IBD [11, 12]. The combination of these factors induces abnormal innate and adaptive immunological responses that threaten the intestinal barrier integrity, cumulatively leading to systemic fallout and malfunctioning of the gut-brain axis [10]. Although IBD is an idiopathic disease affecting the GI tract, both human and animal studies have found a significant correlation between intestinal inflammation and psychological disorders [13]. Depression is as high as 21–27% in patients with IBD compared to 12–13% in healthy controls with the rate of depression rising to 35% during active IBD, with no notable differences between CD and UC pathologies [14, 15]. A postulated aetiological trigger of depressive symptoms in IBD patients suggests systemic low-grade neuroinflammation [16]. It has been reported that neuroinflammation induces one or more of the following: (1) dysregulation of the hypothalamus–pituitary–adrenal (HPA) axis [17], (2) depletion of serotonin levels [18], and (3) alteration of neurogenesis in the hippocampus [19], all of which involved in major depressive disorder (MDD) [16]. Moreover, neuroinflammation-associated depressive symptoms may involve systemic immune factors as an etiological trigger. This has been supported by (i) high levels of pro-inflammatory cytokines in the circulation are seen in patients with MDD [20], (ii) disease treatments requiring exogenous administration of cytokines evoke psychiatric changes [21], (iii) in animals and humans, administration of lipopolysaccharide (LPS) accompanied by the release of pro-inflammatory cytokines provokes depressive symptoms referred to as sickness syndrome [22], (iv) peripheral inflammatory diseases such as rheumatoid arthritis, obstructive pulmonary disease type 1 and diabetes are often comorbid with depression [6–8]. It has been postulated that neuroinflammation-induced depression in IBD involves peripheral inflammatory mediators originating from the inflamed gut penetrating the BBB and either directly or indirectly activating the resident macrophage-like microglial cells within the central nervous system (CNS) [16, 23,24,25]. Activated microglial cells can produce enzymes and mediators that deplete serotonin availability, impair maturation and proliferation of hippocampal progenitor cells and promote neurodegeneration [26, 27].

This review explores in detail the structural and physiological alterations in the GI tract, blood circulation and CNS in IBD patients and corresponding animal models of IBD. The aim is to provide a link between the gut and the brain with a special focus on circulating immune factors and expose neurobiological and/or immunological overlap between MDD and IBD to elucidate an etiological framework for IBD comorbid with depression.

### Intestinal barrier dysfunction in IBD

Structural changes to intercellular and intracellular proteins of the intestinal epithelium and significant alterations of intestinal mucous production imply dysfunctional intestinal barrier integrity in IBD patients enabling luminal antigens to penetrate and initiate local immune responses within the lamina propria [28, 29].

#### The intestinal mucosa and epithelium

**Fig. 1:**
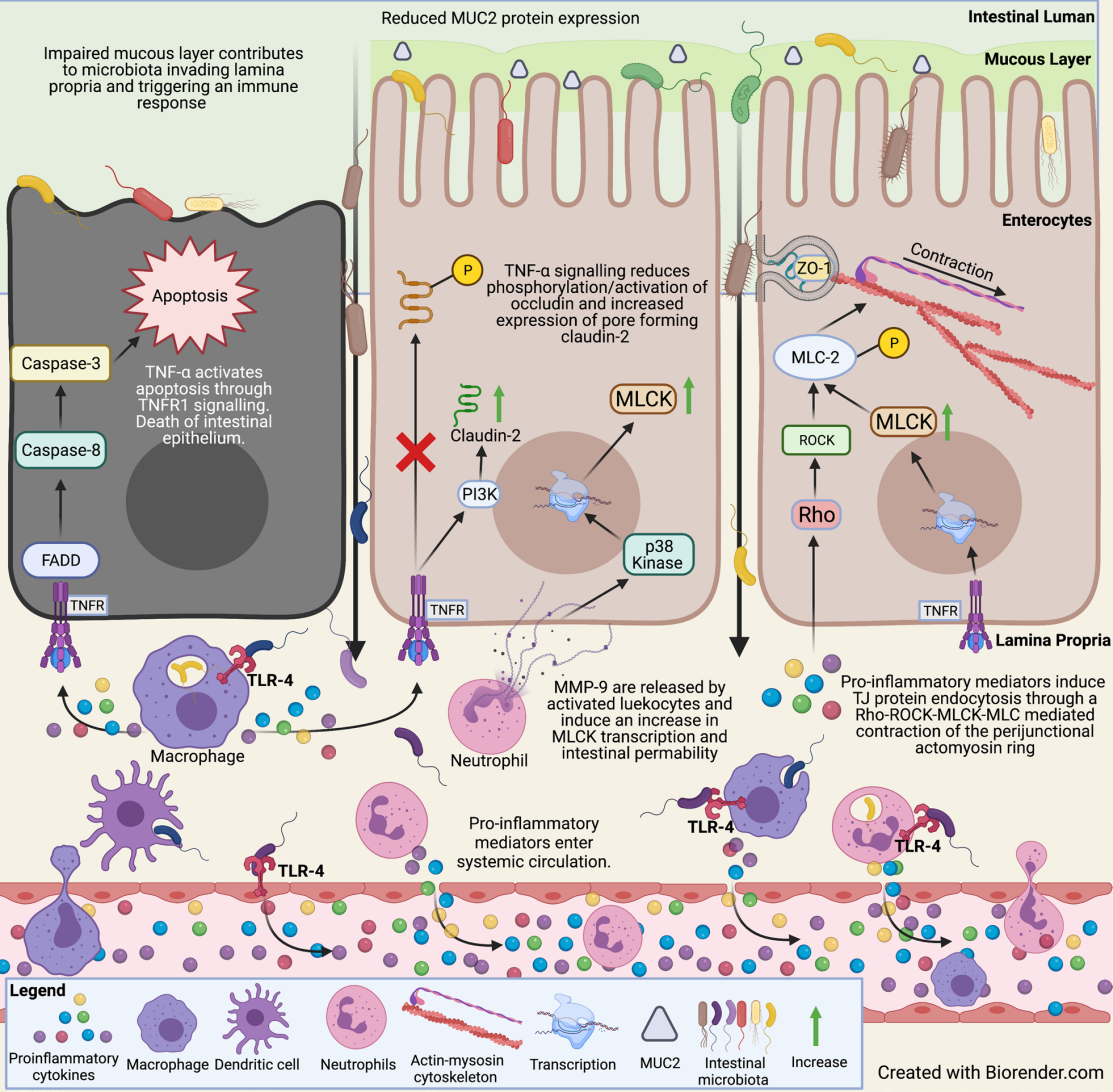
Schematic overview of the mechanisms underlying intestinal barrier dysfunction commonly seen in human IBD and animal models of colitis. Impaired mucous production and composition and/or impaired tight junction protein localisation and production result in luminal microbiota and toxin paracellularly translocating into the intestinal lamina propria layer. Immune cells in this region interacting with antigens trigger the production of inflammatory mediators, which facilitate the recruitment of other leukocytes and lymphocytes. Inflammatory mediators enter peripheral circulation whereby they may trigger distant immunological activation. *FADD* Fas-associated protein with death domain; *IFN* Interferon; *IL* Interleukin; *MLC* myosin light chain; *MLCK* myosin light-chain kinase; *MMP* metalloproteinase, *P* phosphate; *PI3K* phosphoinositide 3-kinase; *ROCK* Rho associated protein kinase; *TJ* tight junction; *TLR* toll-like receptor; *TNF* tumour necrosis factor; *ZO* zonula occludens

The intestinal barrier includes a thick secreted hydrated mucus layer which provides a physical and chemical barrier against luminal microbiota and antigens, as well as lubricating the epithelium [28]. The epithelial barrier is composed of several different classes of intestinal epithelial cells involved in regulating and maintaining barrier functions [28]. These cells include the goblet and Paneth cells, which synthesize and produce the mucin glycoproteins and some anti-microbial proteins, whose synthesis in Goblet cells is under control of ENS produced IL-18 [28, 30]. Mucin proteins such as MUC2 provide the mucus layer with viscous properties [31] and enable the mucus to retain antimicrobial proteins such as defensins, cathelicidins, lysosomes, and immunoglobulins (Ig) such as soluble IgA, IgG, and IgM [28]. Patients with CD show goblet cell hypertrophy as expected with increased mucus formation and a moderate increase in expression of MUC2 and MUC3, and high expression of MUC4 [32]. UC patients exhibit a reduction in the number of goblet cells, MUC2, MUC3, and MUC4 resulting in a diminished mucosal barrier [32] (Fig. 1).

Although a dysfunctional mucus layer is observed in patients with IBD, in vivo animal models of colitis have revealed conflicting results. The *Math*1 gene, also known as the Atonal homologue 1, is a transcription factor involved in the differentiation of goblet cells and Paneth cells [33]. Use of an in vivo murine model of intestinal *Math1* knockout demonstrated that 75–90% loss of secretory cells in the crypts and villi did not generate spontaneous colitis [34]. Moreover, transgenic mice possessing the toxic diphtheria gene, driven by the murine intestinal trefoil factor promoter that facilitated targeted ablation of goblet cells, had a decreased body weight loss and mortality rate (5% vs 55%) compared to non-transgenic mice following administration of dextran sulfate sodium (DSS) [35]. These findings suggest increased resistance to chemical administration of DSS following goblet cell reduction. These studies contrast with findings in the *Winnie* mouse model of spontaneous chronic colitis, which possesses a missense mutation on the *Muc2* gene resulting in robust intestinal inflammation with a phenotype and characteristics similar to UC patients [36, 37]. Histological and immunological presentation include increased intestinal production of pro-inflammatory cytokines, epithelial dysfunction, and endoplasmic reticulum stress within goblet and Paneth cells likely via aberrant folding and assembly of the mucin complex [36, 37]. These paradoxical in vivo findings require further investigation and suggest that a combination of normal mucous secretion and protein composition is a key to facilitating healthy physiological function. Overall, perturbations in mucous production are involved in robust inflammatory responses, which can eventuate in intestinal epithelial barrier dysfunction and may lead to invasion of intestinal contents and/or inflammatory mediators into blood circulation.

#### The intestinal tight junctions

The single-cell layer of the epithelium relies on paracellular protein junctional complexes such as tight junctions (TJs), adherens junctions, and desmosomes for structural integrity and cohesion [38]. TJs are on the apical side of the epithelial cells and provide a boundary between the basolateral and apical membranes [38]. TJs consist of transmembrane proteins such as claudin, occludin, junctional adhesion molecule (JAM), and tricellulin which interacts with peripheral membrane linker proteins such as zonula occludens (ZO) and cingulin which bind to cytoskeleton proteins including F-actin and myosin [38]. The TJ protein complex acts like a “gate” which restricts paracellular entry of large hydrophilic molecules [38]. Altered TJ patterns have been observed in both IBD patients and animal models of intestinal inflammation with their dysfunctions enabling entry of luminal antigens into the lamina propria triggering inflammation [38]. Claudin proteins consist of 27 isoforms, which can be subdivided based on their functional roles [39]. Claudin-2 has been described to increase paracellular permeability, whereas claudin-1, -3, -4, -5, and -8 provide barrier strengthening properties for the cells of the epithelium [39, 40] (Table 1). The different claudin isoforms have been found to be both up and downregulated in the inflamed intestine from patients with IBD [39, 40] (Table 1). Dampened expression for claudin-3, -4, and -7 and increased expression of claudin-1 and 2 are observed in the intestinal epithelium of UC patients [40,41,42,43] (Table 1). Similarly, patients with CD have reduced expression of claudin-3, -4, -5, and -8 proteins with an increased claudin-1 and -2 intestinal epithelial expression [40, 44] (Table 1).

**Table 1:** A comparison of tight junction expression in patients with CD and UC and experimental models of colitis

Junction complex protein	Function	Human IBD		Mouse models of colitis	
		CD	UC	TNBS	DSS
Claudin-1	Decreases paracellular permeability [39]	↑ [45]	↑ [43]	↓ [46] [47]	↑ [48]
Claudin-2	Increases paracellular permeability. Important pore forming protein [39]	↑ [42, 43]	↑ [42, 43]	↓ [49]	↑ [50]
Claudin -3	Decreases paracellular permeability [40]	↓ [42]	↓ [42]	↓ [47]	↓ [48]
Claudin-4	Decreases paracellular permeability [39]	↓ [42]	↓ [42]	–	↓ [51]
Claudin-5	Decreases paracellular permeability [39]	↓ [44]	–	No change [47]	↓ [48]
Claudin-7	Acts as an anion barrier and pore [40]	No change [44]	↓	–	↓ [48]
Claudin-8	Decreases permeability [39]	↓ [44]	–	↓ [47]	↓ [48]
Occludin	Regulates paracellular permeability and cellular adhesion [39]	↓ [52]	↓ [52]	↓ [47]	↓ [53]
ZO-1	Facilitates connection between TJ and intracellular actin cytoskeleton [39]	↓ [54]	↓ [55]	↓ [47]	↓ [46]
MLCK	Phosphorylates MLC causing contraction of peri junctional actomyosin [56]	↑ [57]	↑ [57]	↑ [58]	↑ [59]
Phosphorated (active) MLC	MLC facilitates internalisation of TJ [56]	↑ [57]	↑ [57]	↑ [60]	↑ (59)

↑ upregulated; ↓ downregulated; – no explicit data; *JAM* junctional adhesion molecule; *MLC* myosin II regulatory light chain; *MLCK* myosin light chain kinase

Other TJ proteins like occludins, JAM, and ZO have altered expression patterns in the inflamed intestinal mucosa from IBD patients. Occludin consists of four transmembrane domains and two extracellular loops [38]. Its phosphorylation state on serine and threonine residues determines its cellular localisation and hence TJ stability and permeability. A high phosphorylation state localises occludin in the membrane, whereas decreased phosphorylation correlates with cytoplasmic localisation [38]. Moreover, the occludin promoter can be downregulated by pro-inflammatory cytokines such as tumour necrosis factor (TNF)-α and interferon (IFN)-γ [61]. IBD patients show decreased occludin protein and mRNA expression in the colonic mucosa, which may reflect occludin modulation via inhibition of its promoter [52, 61]. Treatment of the intestinal epithelial model of colorectal adenocarcinoma cells-2 (Caco-2) with TNF-α diminished expression of activated phosphorylated occludin which resulted in increased transepithelial permeability [62] (Fig. 1). The transmembrane TJ protein JAM has also been implicated in TJ dysfunction observed in colitis [63]. Reduced JAM-A protein expression at the level of the intestinal epithelium corresponding with enhanced intestinal permeability was observed in IBD patients [63]. Additionally, mice subjected to DSS-induced colitis in vivo following JAM-A deletion had an increased incidence of severe colitis and showed enhanced intestinal permeability [63]. It has been established that in vitro co‐stimulation of epithelial cells with INF‐γ can induce the internalization of JAM-A [64].

Additionally, TNF-α treatment of Caco-2 cells has profound effects on the linker protein ZO [65]. TNF-α induced downregulation and altered localization of ZO-1 protein, accompanied by an increase in epithelial permeability in vitro [65]. It was found that IFN-γ affects ZO-1 and occludin protein expression via the adenosine monophosphate-activated protein kinase-dependent pathway [54]. ZO proteins anchored to the cytoskeleton actomyosin ring can facilitate TJ contractions and endocytosis resulting in increased intestinal permeability [56]. Myosin light chain kinase (MLCK) and Rho-associated coiled containing protein kinase (ROCK) phosphorylate myosin light chain (MLC) causing contraction of peri-junctional actomyosin rings [56] (Fig. 1). Increased activation of RhoA/ROCK has been detected in inflamed colonic mucosa from patients with CD and the rat model of trinitrobenzene sulfonic acid (TNBS)-induced colitis [66]. Intestinal tissue samples from patients with IBD have increased ileal epithelial MLCK and increased colonic expression of phosphorylated MLC-2 [56, 57]. These findings in IBD patients may be due to increased local production of TNF-α which increases MLCK1 synthesis [67]. Moreover, numerous pro-inflammatory cytokines implicated in IBD such as IFN-γ, TNF-α, and interleukin (IL)-1β can induce TJ protein endocytosis through a Rho-ROCK-MLCK-MLC mediated contraction of the peri-junctional actomyosin ring [68,69,70] (Fig. 1). Matrix metalloproteinases (MMPs)-9 synthesis and secretion are significantly induced after exposure to the cytokines (TNF-alpha, IL-1 alpha) and MMP-9 has been implicated in TJ epithelial dysfunction via a p38 kinase signal transduction pathway [71,72,73] (Fig. 1).

### ENS and intestinal permeability

The ENS is believed to play a role in modulating intestinal permeability through the release of neurotransmitters and via the secretion of peptides and lipids by enteric glial cells (EGC) [74, 75]. The ENS is the largest division of the autonomic nervous system (ANS) and consists of a mesh-like system of neurons that influences GI functions such as segmentation, peristalsis, and secretion [74]. Importantly, the ENS is capable of acting independently of the sympathetic and parasympathetic nervous systems but can be modulated by them under certain circumstances [74]. The ENS can be subdivided into two major nerve plexi: the myenteric plexus, which functions to provide motor innervation to the longitudinal and circular muscles and to coordinate motility and secretion, and the submucosal plexus, which regulates secretion, vasodilation and probably has a role in immune responses [75].

Abnormalities of the ENS such as axonal damage and necrosis, neuronal death, and hyperplasia of EGCs are seen in IBD [76]. This affects normal gut functioning controlled by the ENS, which includes maintenance of the intestinal barrier.

Acetylcholine, endocannabinoids, neuropeptide Y (NPY), and VIP released by enteric neurons have been shown to alter intestinal permeability [77,78,79]. NPY is implicated in upregulation of the pore-forming claudin-2, which can increase intestinal permeability [80]. This is believed to be facilitated through a phosphatidyl inositol-3-kinase (PI3K) pathway and influenced by TNF given that TNF inhibitors reduce expression of NPY [80]. It is speculated that NPY and TNF may operate bidirectionally as in vivo studies in mice with complete knockout of NPY showed reduced secretion of TNF by enteric neurons accompanied by reduced intestinal permeability [80].

Cholinergic pathways are also implicated in intestinal permeability. The cholinergic agonist, nicotine, prevented an increased Caco-2 permeability produced by exposure to cytomix which consists of cytokines known to increase intestinal permeability including TNF-α, INF-γ, and IL-1β [81]. Nicotine-induced barrier preservation is believed to be due to nicotine-activated EGCs, which prevents increased phosphorylation of IκBα and NF-κB expression [81]. Primary cultures of porcine colonocytes exposed to the cholinergic agonist, carbachol, and the muscarinic agonist, oxotremorine, demonstrated enhanced transepithelial electrical resistance indicative of increased epithelial tightness [82].

Vasoactive intestinal polypeptide (VIP) may play a role in decreasing intestinal permeability following electrical field stimulation of submucosal neurons in vitro [79]. These findings were accompanied by enhanced expression of ZO-1 [79]. Impaired VIP signalling was observed in (TNBS)-induced colitis which is associated with a dramatic reduction of slow excitatory synaptic transmission in VIP-expressing secretomotor neurons in the submucosal plexus of guinea-pig [83]. Moreover, using sterically stabilized micelles for VIP administration (VIP-SSM), it was observed that a single dose of VIP-SSM significantly improved histological score, alleviated diarrhoea, and decreased pro-inflammatory cytokines in mice with DSS-induced colitis [84]. It could be theorised that reduced VIP levels may be in part responsible for impaired intestinal barrier noted in IBD patients, so VIP may be a useful therapeutic target in the future.

Cannabinoid signalling is an important mechanism of synaptic modulation in the nervous system and is believed to play a role in the intestinal barrier. Exogenous cannabinoids and endocannabinoids act on the G-protein coupled cannabinoid receptor (CBR) 1, which predominantly exists on nerve terminals where they may modulate neurotransmitter release, and on CBR2, which are found mainly on immune cells where they can mediate immune suppression [85]. CBR1 has been localized on myenteric neurons of the rat and guinea-pig intestine where nearly all cholinergic neurons express CBR1 and has been shown to preserve intestinal barrier integrity [86]. CBR1^−/−^ mice exposed to stressful stimuli had enhanced expression of pro-inflammatory enzymes including cyclooxygenase-2 (COX2) and NOS2, increased colonic permeability to chromium-51-labelled ethylenediaminetetraacetic acid, and enhanced translocation of bacteria to the mesenteric lymph nodes compared to stressed wild type mice [78]. Degradation of barrier function was postulated to be due to NO-induced cytoskeleton rearrangement and subsequent tight junction dysfunction since in vitro and in vivo findings of NOS2 activity promoted intestinal epithelial permeability through NO synthesis [78, 87, 88].

The enteric glial population has a vital role in maintaining mucosal barrier function. Transgenic mice with targeted ablation of EGCs have a disrupted mucosal barrier and resultant inflammation with enhanced mucosal paracellular permeability to small fluorescent probes [89]. Moreover, Caco-2 cells co-cultured with enteric glia showed significantly greater transepithelial resistances and diminished permeability to fluorescein isothiocyanate (FITC)-dextran and fluorescein sulfonic acid. This correlated with a significant up-regulation of ZO-1 and occludin as increased F-actin accumulation to lateral membranes [89]. These findings may be explained by EGC-derived neurotrophic factors, such as glial-derived S-nitrosoglutathione (GSNO) and glial cell line-derived neurotrophic factor (GDNF), which have been implicated in altering intestinal permeability [89,90,91,92]. GSNO administration in vitro and in vivo restored the appropriate localization and expression of ZO-1 and occludin, F-actin accumulation to the lateral membranes, as well as reduced phosphorylation of MLC in the intestinal epithelium [89, 91, 92]. Moreover, GSNO attenuated enhanced intestinal permeability induced by cytomix and LPS in Caco-2 cell cultures and rats, respectively [91, 92]. GSNO may influence tight junctions through S-nitrosylation of inhibitory κB kinase (IKK), which prevents phosphorylation of the inhibitor of κB (IκB) [91, 92]. Interestingly, higher concentrations of GSNO have been shown to impair epithelial barrier function in vitro characterised by a marked disruption of the F-actin network [89]. Biopsy samples from CD patients, which trend toward higher mucosal permeability compared to controls, showed a significant reduction in permeability to FITC-inulin following the addition of GSNO [89]. This may suggest that the EGC network may be disrupted in intestinal mucosa of CD patients, resulting in lower tissue GSNO concentration. Lower concentrations of GSNO may impair tight junction expression and enhance intestinal permeability through an NFκB pathway [91, 92]. EGCs have been shown to be the main source of GDNF, which affects gut barrier properties [93]. GDNF administration to immature intestinal cell lines promoted linearized and augmented staining patterns of the tight junction proteins occludin and claudin-1, 5 at the cell borders as well as enhanced epithelial proliferation and decreased permeability assessed by FITC-dextran and transepithelial electrical resistance (TEER) [90]. Administration of GDNF in vitro or co-culture with EGCs reduced downregulation of tight junctions in rat intestinal epithelial cells and prevented the drop in TEER following ischemia–reperfusion injury [94]. Moreover, EGCs significantly increase GDNF expression when stimulated by hypoxia-reoxygenation [94]. Furthermore, GDNF has a potent anti-apoptotic effect on colonic epithelial cells via activation of both mitogen-activated protein kinase (MAPK) and PI3K/AKt signalling pathways [95, 96]. Expression of GDNF and glial marker glial fibrillary acidic protein (GFAP) is significantly higher in inflamed colonic biopsies from UC patients than in healthy controls [97]. This may be due to enhanced pro-inflammatory cytokines being effective stimuli for GDNF secretion [93]. In contrast, reduced GFAP and GDNF expression is noted in CD patients [97]. In rats with DSS-induced colitis, recombinant adenoviral vectors encoding GDNF administered via the rectum significantly ameliorated the severity of inflammation [95].

Thus, the ENS plays a vital role in the maintenance of the intestinal barrier keeping the sterile lamina propria free of immunoreactive luminal antigens. During intestinal inflammation, the ENS may become damaged and lose its capacity to maintain the intestinal epithelial barrier contributing to impaired tight junction regulation, mucosal cell regeneration, and the invasion of luminal microbiota into the immune-rich lamina propria. Consequently, this helps facilitate entry of systemic inflammatory mediators into circulation, which may influence CNS neurobiology and mood states.

#### Intestinal endothelial dysfunction

The consequence of impaired epithelial integrity, mucus production and ENS dysfunction, is a translocation of luminal exogenous factors such as microbiota, toxins, and antigens into the lamina propria [98]. As a result, a robust inflammatory response facilitates the uptake of inflammatory mediators into peripheral circulation [99]. Following penetrations into lamina propria, luminal antigens are recognised by pattern recognition receptors such as toll-like receptors (TLR), nucleotide-binding and oligomerization domain, and C-type lectin receptors triggering the activation of a nuclear transcription factor NF-*κ*B and inflammasomes [100]. This elicits the production of pro-inflammatory cytokines in the local tissue, including IL-1*β*, IL-6, TNFα, IFN-*γ*, and cytokines involved in the IL-23/Th17 pathway [100, 101]. Inflammation leads to endothelial cell dysfunction and therefore may facilitate translation of pro-inflammatory mediators from gut to the peripheral circulation [102, 103]. Human intestinal microvascular endothelial cell cultures can produce different cytokines (IL-1β, IL-3, and IL-6) on stimulation with pro-inflammatory cytokines such as TNF-α and IL-1 [103]. Additionally, gut endothelial cells constitutively express TLR5 on their basolateral surface [104]. The binding of flagellin, a prominent antigen in IBD, can induce these endothelial cells to produce pro-inflammatory cytokines and adhesion molecules [103, 105] (Fig. 1).

Many anti-inflammatory cytokines have been implicated in the pathogenesis of IBD and have varying implications in endothelial functions, including transforming growth factor beta (TGF-β) and IL-10. TGF-β secretion was found enhanced in lamina propria localised mononuclear cells in UC patients but decreased in CD patients [106]. Moreover, the expression of TGF-β and its receptors was increased in intestinal cells of patients with IBD [107]. TGF-β can have a detrimental effect and contribute to intestinal fibrosis in IBD patients which worsens disease outcomes [108]. In the context of endothelial function, TGF-β has been suggested to increase endothelial permeability through activin receptor-like kinases (ALK) receptors 5 [109, 110]. TGF-β and ALK5 ligation is believed to activate TGF-β induced ALK5 signalling, which leads to phosphorylation of Smad2 and Smad3, inhibition of angiogenesis, and increased endothelial permeability [109, 110].

Studies have indicated reduced IL-10 expression is a pathophysiological trait in IBD and an inducer of increased vascular permeability [111, 112]. An IL-10 knockout mouse model of colitis shows increased endothelial permeability assessed by monolayer electrical resistance, increased albumin permeability, and reduced expression of occludin [112]. Moreover, endothelial cell dysfunction in IL-10 knockout mice is mediated by IFN-γ activity, suggesting that endothelial barrier permeability is regulated reciprocally by IL-10 and INF-γ [112].

Additionally, endothelial dysfunction corresponds with infiltration of leukocytes such as neutrophils and monocytes [103]. Accumulation of intestinal neutrophils and monocytes in the lamina propria induces release of mediators that jeopardise endothelial junctions via protease secretion and upregulation [103]. For instance, neutrophil-derived elastase proteins are elevated during IBD pathogenesis and can degrade endothelial junctional proteins such as cadherin [103, 113]. Overall, interactions between inflammatory mediators and gut antigens likely to enhance endothelial permeability and/or production of pro-inflammatory cytokines contributing to significant increases in systemic circulating inflammatory mediators. Serological studies have confirmed elevations in many immune mediators in the serum of IBD patients [114, 115]. Evidence of circulating immune factors in the serum of patients with IBD is important given that it provides a route by which the gut can modulate distant sites such as the brain, which may induce mood disturbances such as depression.

### Humoral response in inflammatory bowel disease

Several studies investigated serological cytokine signatures in paediatric patients with IBD in order to identify inflammatory biomarkers in the blood for diagnosing and evaluating IBD. Analytes included IL-13, IL-1β, IL-4, IL-6, INF-γ, TNF-α, IL-1 receptor antagonist, IL-12, IL-8 IL-5, IL-7, CCL11, IFNγ-induced protein 10 (IP-10), macrophage inflammatory protein, granulocyte–colony-stimulating factor, and fibroblast growth factor (FGF) which were detected in sera acquired from IBD patients compared to healthy controls [114, 115]. Plasma infiltration of LPS with endotoxemia is present in 48% of CD patients and 28% of UC patients [116]. Moreover, sera levels of LPS and 1,3-β-D-glucan were found to be increased in patients with active CD compared to those in remission and controls, with sera TNF-α correlated with LPS and 1,3-β-D-glucan [117].

These studies provide a fundamental understanding of the biomarker signatures for IBD. However, inflammatory mediators have been postulated to induce systemic fallout resulting in other system compromises, including damage to the blood–brain barrier (BBB) in patients with IBD [118].

#### Cytokine-induced damage to the blood–brain barrier

**Fig. 2:**
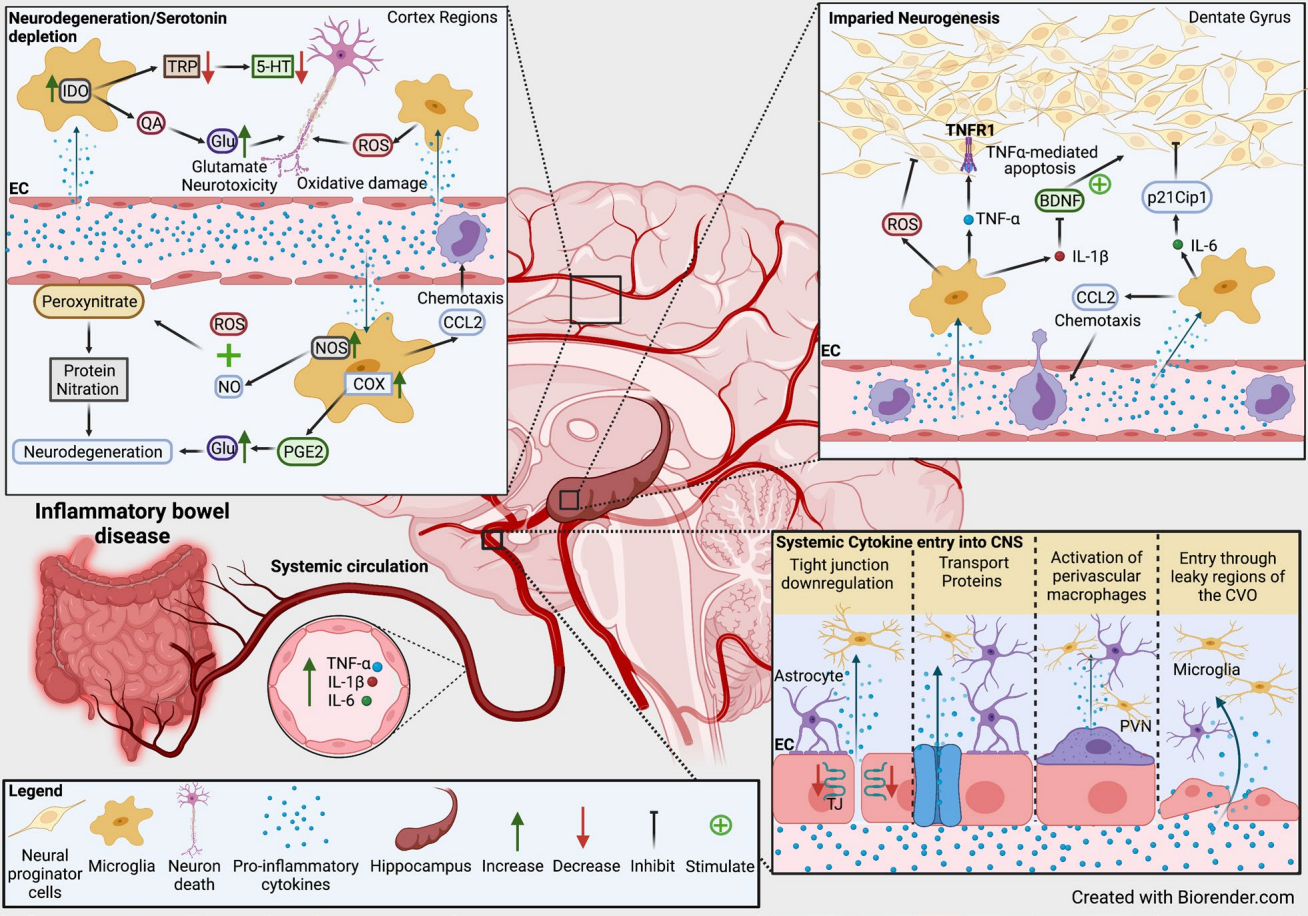
Schematic diagram of neuroinflammatory changes seen in and postulated in human and animals with intestinal inflammation. Circulating inflammatory mediators enter into brain parenchyma through the suggested mechanisms whereby they may modulate local glia populations such as the microglia. Microglia can impact the various neurobiological correlates of depression including neurodegeneration, serotonin biosynthesis, and hippocampal neurogenesis. *5-HT* 5-hydroxytryptamine (serotonin); *BDNF* brain-derived neurotrophic factor; *CCL2* chemokine (C–C motif) ligand 2; *CNS* central nervous system; *COX* cyclooxygenase; *CVO* circumventricular organ; *EC* endothelial cells; *Glu* glutamate; *IDO* indoleamine-pyrrole 2,3-dioxygenase; *IL* interleukin; *NO* nitric oxide; *NOS* nitric oxide synthase; *PGE2* prostaglandin E2; *PVN* perivascular macrophages; *QA* quinolinic acid; *ROS* reactive oxygen species; *TJ* tight junction; *TNF* tumour necrosis factor; *TNFR1* tumour necrosis factor receptor-1; *TRP* tryptophan

Serological inflammatory mediators seen in IBD and animal models of colitis may impede TJ regulation in brain endothelial cells ultimately leading to a dysfunctional BBB marked by enhanced permeability [119]. In vivo and in vitro studies have shown that circulating cytokines can under some circumstances modulate expression of TJ proteins in cerebral endothelial cells [120,121,122,123] (Fig. 2). For instance, IL-1β has been shown to suppress astrocytic sonic hedgehog (SHH) production [123]. In vitro, using a SHH conditioned media, SHH, or an SHH signal agonist strengthens the BBB integrity by upregulation tight junction proteins, including claudin-5, ZO-1, and occludin [123]. These effects were abrogated by a SHH signal inhibitor [123].

Conversely, in vivo IL-10 attenuated the increased BBB permeability in rat models of severe acute pancreatitis by reducing brain microvascular endothelial cells apoptosis through a signal transducer and activator of transcription 3 (STAT3) pathway mediated downregulation of claudin-5 expression [124]. Similarly, IL-25 has been shown to preserve BBB and is expressed by brain capillary endothelial cells (BCECs) [125]. In vitro, IL-25 is downregulated by many pro-inflammatory cytokines, including TNF-α, IL-1β, and IL-6 [125]. IL-25 has been shown to restore the reduced expression of tight junction proteins, occludin, JAM, and claudin-5, induced by TNF-α in BCECs, leading to the restoration of TNF-α-induced BBB permeability [125]. Cytokine-induced regulation of BBB permeability may explain findings in animal models of colitis. A reduction in occludin and claudin-5 observed in the hippocampus and cortex of DSS-treated mice was suggested to be due to elevated serum IL-6 levels [126]. Increased BBB permeability to tracers in animal models of intestinal inflammation may reflect modulation of cerebral endothelial cells by serologic immune factors [127, 128]. A significant increase in BBB leakage predominantly in and around the circumventricular organs and focal areas of the brain parenchyma indicating impaired BBB integrity was observed in TNBS-induced colitis in Sprague–Dawley rats [128]. Moreover, enhanced infiltration of fluorescein, but not FITC-dextran, showing increased BBB permeability to smaller molecules, was observed in rabbits with either acetic acid or TNBS-induced colitis [127]. Importantly, this study confirmed that intestinal inflammation, not the treatment method, conferred BBB permeability given that both treatments enhanced permeability. Moreover, colitis has been associated with decreased transcription of ZO-1 (*Tjp1*) and claudin-5 (*Cldn5*) in the brain [129]. Transcription of TNF-α, IL-1β, and IL-6 correlated with decreased transcription of *Tjp1*, but not *Cldn5* which could indicate that these cytokines may increase BBB permeability through ZO-1 downregulation [129].

Overall, these findings provide evidence that circulating pro-inflammatory cytokines result in injury to the endothelial cells of the BBB. Moving forward, diminished BBB integrity predisposes translocation of circulating neuroinflammatory mediators into brain parenchyma, which may affect the neuroglial networks and their regulation in the various regions of the brain.

#### Cytokine entry and inflammatory responses in the central nervous system

Circulating inflammatory mediators derived from the inflamed gut penetrate the brain following BBB dysfunction as noted in animal models of colitis. Circulating inflammatory mediators also affect the CNS through other mechanisms. For instance, they (i) enter through “leaky regions” in circumventricular organs [130] (Fig. 2), (ii) activate peripheral vagal nerve afferents that relay cytokine signals to the nucleus of the solitary tract and hypothalamus or HPA axis [131, 132], (iii) activate and induce the release of local inflammatory mediators by endothelial cells and perivascular macrophages in the cerebral vasculature [133] (Fig. 2), (iv) induce activation and diapedesis of peripheral monocytes/macrophages and T lymphocytes into the brain parenchyma [134], and (v) through the utilisation of endothelial transporter protein channels [119] (Fig. 2). Whether through the listed mechanisms or via BBB dysfunction, inflammatory mediators may penetrate and modulate local CNS glial cells. Indeed, evidence of local immune activity in the brains of animals with colitis has been found [126, 135, 136].

### Neuroinflammation in the CNS during colitis

#### Inflammatory markers associated with neuroinflammation in colitis

Several studies in animal models of colitis have identified inflammatory markers in the hippocampal and cortical brain regions [126, 135,136,137,138,139,140,141,142,143]. There is a significant increase in IL-1β and IL-6 mRNA expression in the cortex and IL-1β and TNF-α in the hippocampus of mice with DSS-induced colitis [126]. This is accompanied by significantly higher serum levels of IL-6 and TNF-α in mice with colitis [126, 135]. Moreover, using TNBS-induced model of colitis in rats, Wang et al. (2010) reported intestinal morphological damage, increased myeloperoxidase activity, and increased mRNA and distribution of IL-6 in the inflamed colon and specific regions of the brain including the cerebral cortex and hypothalamus [144]. Neuroinflammatory changes are considered to be an indicator of alterations in animal behaviour in in vivo models of IBD [136]. In mice with dinitrobenzene sulfonic acid (DNBS)-induced colitis, a significant increase in the expression of TLR-2 and -4, TNF-α, IL-6 and damage-associated molecular patterns like high mobility group box protein 1 (HMGB1), intracellular signalling proteins such as myeloid differentiation primary response 88, and brain-derived neurotrophic factor (BDNF) was found in the hippocampal regions [136]. Enhanced innate immune responses in the brains of animal models of colitis have been associated with depressive behavioural traits seen as decreased mobility time in the forced swim and tail suspension tests, decreased grooming in the splash test and sucrose intake in the sucrose preference test [136]. Moreover, the inflammatory activity associated with anxiety and depression in mice with colitis was accompanied by alterations to hippocampal mitochondrial parameters [136]. These include decreased antioxidant glutathione (GSH) and adenosine triphosphate levels together with overproduction of reactive oxygen species (ROS) (Fig. 2), suggesting mitochondrial dysfunction and possible oxidative stress in the hippocampus of mice with colitis [136].

These findings are pertinent in IBD-associated depression given that brain metabolism impairments characterised by mitochondrial dysfunction and the generation of ROS have been implicated in the pathogenesis of depression and anxiety [145, 146]. Moreover, GSH, a major brain antioxidant that ameliorates oxidative species, is reduced in the prefrontal cortex of MDD patients [147].

In another study, mice with TNBS-induced colitis showed heightened behavioural despair and increased hippocampal TNF-α, inducible nitric oxide synthase (iNOS), and nitrite expression [137]. In this study, only male mice were used in order to remove the confounding variable of high estrogen in females given its correlation with elevated serum cytokine production in chemically-induced colitis [148]. Future studies should determine the influence of estrogen on neuroinflammatory changes associated with colitis. Elevated iNOS activity in the hippocampus region and associated behavioural despair in this study [137], may suggest elevated nitric oxide (NO) production. NO is thought to play a central role in the neurobiology of depression [149]. In line with this, iNOS-inhibitors reduce behavioural despair of mice with colitis [137]. NO-associated depression may be due to impaired neurotransmitter synthesis and/or neurodegeneration [150, 151]. However, currently no studies in animal models of colitis have elucidated this mechanism. Indeed, in rats inhibition of NOS elevates levels of extracellular serotonin and dopamine in the ventral hippocampus, a major brain region correlated with depression [150]. During inflammatory pro-oxidant states, excessive NO in the brain can combine with superoxide anions to create peroxynitrate which induces neural degeneration and cell death via protein nitration [151] (Fig. 2). Given that there is evidence of ROS production and oxidative damage in animals with colitis [136, 137], further studies should investigate CNS changes indicative of oxidative damage in animals with colitis.

In a recently published study, DSS-induced colitis found upregulation of inflammatory-related proteins S100A8, S100A9 (also known as myeloid related protein (MRP) 8 and MRP14), and lipocalin-2 (Lcn2, also known as neutrophil gelatinase-associated lipocalin) in the brain. Though S100A9 and Lcn2 are upregulated in colitis, this is the first study to observe evidence of these proteins in the brain of mice with colitis [138]. Neutrophils, macrophages, and monocytes are the main source of these proteins, although other cells can also release them during infection [152]. The S100A8 and S100A9 proteins form a heterodimeric complex S100A8/A9 (also known as calprotectin), which has antimicrobial activity by sequestering trace metals essential for bacterial growth [152, 153]. Following inflammatory stimuli, these proteins are significantly upregulated and released into the extracellular environment, where they can activate immune and endothelial cells [152, 154]. Up regulation of S100A8 and S100A9 has been observed in many inflammatory diseases [155,156,157,158], and faecal calprotectin is used as a marker for IBD severity as its level significantly correlates with intestinal inflammation [153, 159]. Paquinimod, an orally active immunomodulatory quinoline-3-carboxamide derivative, which blocks the interaction of S100A9 with TLR4 and receptors for advanced glycation end products (RAGE), prevented upregulation of Lcn2 and S100A8/A9 in the brain and S100A8/A9 in the colon and ameliorated symptoms of colitis [138, 153, 160, 161]. The upregulation of S100A8 and S100A9 in the brain could be related to the infiltration of peripheral inflammatory cells into the brain (monocytes and neutrophils) observed in this study [138]. However, S100A8 and S100A9 upregulation may be stressed-related as mice with chronic stress have upregulated genes encoding these proteins in the hippocampus [162].

Whilst S100A8 and S100A9 may play a central role in propagating neuroinflammation in colitis models, it has been suggested that NLR family pyrin domain containing 3 (NLRP3) protein activation may be involved [139]. NLRP3 largely functions as an intracellular sensor that detects microbial motifs, endogenous danger signals and environmental irritants [163]. Activation of NLRP3 results in the assembly and activation of the NLRP3 inflammasome, which leads to cleaving of inactive pro-IL-1β and pro-IL-18 into their active forms via caspase 1 [163, 164].

Increased NLRP3 inflammasome activity, microglial and astrocyte activation in the hippocampus and cortex, accumulation of gut-derived T cells along meningeal lymphatic vessels observed in the brains of wild type mice with DSS-induced colitis, were not found in NLRP3 knockout mice [139]. These findings could be the results of a NLRP3 facilitating dysfunction of the “glymphatic” system. The glymphatic system facilitates the entry of subarachnoid cerebrospinal fluid (CSF) into the brain interstitium where it mixes with brain interstitial fluid (ISF) [165, 166]. CSF-ISF then flows through the interstitium, being drained via para venous pathways to the meningeal lymphatic vessels, reaching the cervical lymphatics [165, 166]. Astrocytes allow the movement of fluid between paravascular spaces and the interstitium via water channels such as aquaporin-4 (AQP4), which requires the polarization of AQP4 [167]. This movement of fluid through the brain allows the removal of extracellular proteins, such as amyloid-β peptide (Aβ) and tau, from deeper areas of the brain, where interstitial solutes cannot normally reach the BBB [165, 168]. Impaired glymphatic drainage may lead to Aβ and tau protein accumulation, which has been associated with triggering and propagating neuroinflammation playing a central role in neurodegenerative conditions such as Alzheimer’s disease [169,170,171]. Glymphatic dysfunction, leading to impaired clearance of Aβ and aggravated cognitive decline seen in mice with DSS-induced colitis were attenuated in NLRP knockout mice [155]. This may be due to the binding of IL-1β to cognate receptors on astrocytes leading to the loss of AQP4 polarity [152]. Moreover, this study [139] and others [129, 138, 139] suggest a role of immune cells migrating from the gut to the brain in colitis-induced neuroinflammation. Meninges localised T cells have been shown to infiltrate the CSF, induce microglial activation, and enhance local pro-inflammatory cytokine production [139, 172]. Additionally, other peripheral immune cells have been shown to be elevated in brain samples from animals with colitis including monocytes and neutrophils [129, 138]. Whilst the evidence of neuroinflammation in colitis models is apparent, the underlying mechanisms still require exploration.

#### Microglial cells during neuroinflammation in colitis

After entering the CNS, inflammatory mediators may modulate local neuroglial cells in specific brain regions triggering neuroinflammation in animals with colitis [126, 136]. Among the neuroglial cell populations, microglial cells can migrate and become activated during cytokine-induced neuroinflammation [173]. Microglia are derived from the embryonic mesoderm and are closely related to peripheral macrophages [174]. Functionally, they eliminate cell debris, remove damaged cells and destroy pathogenic agents [175]. Moreover, they support and regulate neurogenesis, maintain oligodendrocyte progenitor cells, neuronal morphology, neural circuitry pathways, and neuronal outgrowth and positioning in the developing brain. During neuroinflammation, the inflammatory milieu activates microglial cells initiating their immunological response [176, 177] (Fig. 2).

Several studies provide evidence that microglia are activated in the brains of mice with colitis [126, 135, 138,139,140,141,142,143]. In DSS-treated mice, significantly higher cortical and CA1 hippocampal immunofluorescence for a microglial marker, ionized calcium-binding adaptor protein-1 (Iba-1), has been observed [126, 141]. Since Iba-1 is a marker of both resting and activated microglia, an increase in Iba-1 immunoreactivity in DSS-treated mice was attributed to a change in microglia morphology and localisation as opposed to increased cell number and consistent with increased microglia reactivity [138]. Increased Iba-1 immunoreactivity may be transient as mice with DSS-induced colitis revealed increased hippocampal Iba-1 expression in acute colitis (day 7 post initial DSS treatment), and showed no difference after chronic colitis (day 29 post initial DSS treatment) [135]. Moreover, DSS administered to weaning (postnatal day 21) mice revealed enhanced gene expression for markers associated with microglia such as *Iba-1, Nos2,* and *IL-1β* along with increased microglia cell numbers, decreased numbers of dendritic processes, and decreased length of processes [140]. However, DSS was administered at the weaning stage, which is a critical point for the maturation of gut microbiota and may be due to gut dysbiosis [140, 178]. Furthermore, rats with TNBS-induced colitis, displayed microglial activation, increased excitability of hippocampal neurons, altered hippocampal glutamatergic transmission, and lowered seizure threshold [142, 143]. An intracerebroventricular injection of anti-TNF-α antibody and minocycline (an inhibitor of microglial/macrophage activation), reversed these findings, which may suggest CNS microglial/macrophage and/or TNF-α involvement in neuroinflammation associated with colitis [142, 143]. In another recent study, the number of microglia was significantly increased in the cortex and hippocampus in DSS-fed WT mice but reduced in the NLRP3 knockout mice [139].

Thus, the proinflammatory cytokines and NLRP3 appear to play a critical role in perturbation in microglia activity in these models. Whether these microglia are activated via mediators originating from the gut is yet to be confirmed and requires more investigation.

#### Microglia activation, migration and neurodegeneration

Increased expression of microglia, suggested in models of colitis, may be due to CNS-derived or circulating cytokines or antigens, which may activate neuroglial cells. As discussed, patients with IBD present with elevated serum levels of many pro-inflammatory cytokines and antigens inclusive of TNF-α, IL-1β, IL-6, and LPS [114,115,116,117]. If BBB dysfunction is indeed found in patients with IBD, these circulating factors may enter the brain parenchyma and alter microglial function conducive to the progression of neuroinflammation. Importantly we see evidence of upregulation of TNF-α, IL-1β, and IL-6 in various brain regions of colitis models [126, 129, 136, 137], which may be sourced from or interact with neurons, microglia, and other cells.

Indeed, in vitro studies demonstrate the capability of cytokines to activate microglial cells and induce their release of pro-inflammatory and neurotoxic mediators [176, 177, 179]. For instance, stimulation of microglia with recombinant TNF-α induces upregulation of many pro-inflammatory mediators such as TNF-α, Nos2, and Il-1β via an NF-kB p65 pathway [176]. As mentioned above, excessive NO may trigger neuronal cytotoxicity through protein nitration [180]. Whether NO neurotoxicity from microglia occurs in the CNS of colitis models is yet to be explored, but, as discussed earlier, TNBS-induced colitis was associated with a significant increase in hippocampal TNF-α and iNOS protein levels which could reflect reactive microglia activity [137]. Furthermore, stimulation of mouse microglial cell line BV-2 with IL-1β induces expression of pro-inflammatory markers such as COX-2, chemoattractant protein-1 (CCL2), and IL-6 via a PI3K/Akt pathway [177]. This pathway and associated inflammatory markers could be relevant in colitis as NLRP3 inflammasome activity (critical for caspase 1-dependent release of IL-1β and IL-18) in the CNS has been implicated in the exacerbation of neuroinflammation by DSS-induced colitis in aging mice [139, 181]. Moreover, increased levels of mRNAs for TNF-α, IL-1β and COX-2 protein expression were found in isolated rat cerebral cortex microglial cell cultures treated with recombinant IL-6 compared to untreated control [179]. COX-1 and -2 catalyse the formation of prostaglandins, thromboxane, and levuloglandins [182]. In vivo, systemic TNF-α and LPS administration activated microglia and increased expression of brain pro-inflammatory factors in WT mice, but not in TNF R1/R2 deficient mice [183]. Indeed, this may be consistent with studies that observed normalisation of synaptic transmission following either anti-TNF-α or minocycline treatment in animals with colitis [142, 143, 184].

Enhanced prostaglandin activity might contribute to the mechanisms involved in the increased BBB permeability observed in models of colitis [126, 127]. Different prostaglandin receptors appear to have varying functions in terms of BBB permeability. In ischemic stroke models, pharmacological or genetic inhibition of PGE2 receptors suggests that EP1 and EP3 receptors contribute to BBB breakdown observed in these models [185,186,187]. EP4 was reported to attenuate BBB dysfunction induced by stroke [188, 189]. However, in animals administered with LPS, prostaglandins show varying effects including blocking, enhancing, or having no effect on the actions of LPS on BBB permeability [190,191,192]. Furthermore, systemic LPS challenge has been shown to induce upregulation of prostaglandin enzyme COX-1 in microglia and perivascular macrophages with PGE2 increase seen primarily in the hippocampus and thalamus [193]. Mice with DSS-induced colitis exhibited more anxiety and less social behaviour than control mice and occurred in parallel with increased circulating IL-6, NPY, and IL-18 levels as well as an increase in hypothalamic *Cox-2* mRNA [194]. In a recent study using DSS-induced colitis, elevated expression of the *Ptgs2* gene, which encodes COX-2, was noted [138].

Systemic LPS challenge in mice elicits the increased amounts of CCL2 mRNA and protein in the hypothalamus and hippocampus, in conjunction with upregulation of chemokine receptor 2 (CCR2) expression by microglia [195]. CCR2 studies in the CNS of mice with colitis are very limited and require further investigation. However, no changes in seizure threshold in colitis mice with impaired CCR2 functioning were found, which suggested that monocytes do not play a major role in colitis-induced neuronal hyperexcitability [129]. CCR2 appears in two isoforms (CCR2A and CCR2B) with CCR2B being the dominant isoform making up 90% of all CCR2 expression and is observed on microglia, astrocytes, and neurons, while CCR2A is observed in certain mononuclear and smooth muscle cells [196,197,198]. CCL2-CCR2 axis can induce the secretion of pro-inflammatory cytokines, such as IL-1β and IL-18 by microglia [197]. Moreover, CCR2 appears critical for microglial accumulation as indicated in CCR2 knockout models [199]. Studies should entice to investigate whether CCR2 is upregulated in brain tissue from animals with colitis, which may help elucidate possible mechanisms underpinning microglial activity seen in animals with colitis. Given that in vitro and in vivo studies evidence the capacity of circulating inflammatory mediators and endotoxins in inducing microglial changes, it may be plausible that alteration in microglia noted in animal models of colitis could be due to systemic infiltration of antigens and immune mediators. Moreover, COX2, PGE2, and CCR2 would be plausible future targets to investigate in the CNS of animals with colitis, given their role in the progression of events relevant to neuroinflammation.

#### Mechanisms of colitis-associated suppression of hippocampal neurogenesis

Impaired hippocampal neurogenesis has previously been associated with microglial cell activity leading to depression and maybe a neurobiological mechanism underlying IBD-associated depression [200]. The association between reduced neurogenesis and depression in humans can only be inferred through reduced hippocampal volume noted in depressed individuals [201]. However, post-mortem cellular changes in depressed humans revealed alterations in the neuropil, altered fluid content, and changes in granule cell and pyramidal cell density. [202]. This may be responsible for hippocampal volume changes in humans. Further research to confirm neurogenesis as a neurobiological correlate of depression is needed.

Adult neurogenesis mainly occurs in the subgranular (SGZ) and subventricular zones in the dentate gyrus of the hippocampus resulting in the formation of new granule cells from neural progenitor cells [203]. Microglia play a vital role in facilitating the complex process of neurogenesis. In vitro studies have demonstrated that microglial conditioned media enhance precursor cell differentiation, neuroblast production, and neuronal survival [204, 205]. In addition, microglia are implicated in eliminating apoptotic neuroblasts and adult neurons through phagocytosis, which is vital given that most of the newborn cells undergo death by apoptosis within the first 1–4 days of their life [206].

Given that increased microglial expression is noted in in vivo animal models of IBD [126, 135,138,139,140,141,142,143], microglia-facilitated impairment of neurogenesis may be responsible for triggering or potentiation of colitis-associated depressive symptoms. Imaging studies show an increase in gray matter volume in the hippocampus of CD patients which may be related to immune activation that induces alterations in glial cells activity [207]. There are limited studies confirming hippocampal dysfunction because of activated microglial cells in animal models of colitis. However, enhanced microglial cell activity in the hippocampus is correlated with a reduction in a neuronal marker, doublecortin (DCX), associated with reduced neurogenesis and behavioural abnormalities in mice with DSS-induced colitis [140]. Moreover, it has been speculated that reduced neurogenesis seen in animals with colitis may be induced by cyclin-dependent kinase inhibitor p21^Cip1^ (p21) activity in the hippocampus. Functionally, p21 restrains cell cycle progression and arrests the cell in the G1 phase [208]. p21 can be induced in early neuronal progenitors and immature neurons in the SGZ and can function to limit these cells’ proliferation and ultimately suppresses neurogenesis [135, 204, 209, 210]. In addition, acute systemic inflammation and pro-inflammatory cytokines, originating from microglia or other cells, can increase p21 expression and restrain hippocampal precursor cells of neuronal lineage in the SGZ [210]. In a study using mice with DSS colitis, acute colitis was correlated with increased p21 expression in the hippocampus [210]. However, in the chronic phase of inflammation, a fourfold increase in p21 mRNA levels was noted [210]. Markers of neuronal stem/early progenitor cells, inclusive of nestin and brain lipid-binding protein, and DCX were downregulated [210]. The nuclear protein Ki-67 and marker of cell proliferation co-labelled with DCX showed a decrease in number during chronic colitis in the SGZ [210].

Microglial-associated cytokines IL-1β, TNF-α, and IL-6 have been shown in vitro to induce p21 expression in differentiating neuronal progenitors and may be partly responsible for the above findings [210] (Fig. 2). Importantly, pro-inflammatory cytokines noted in the hippocampus of animals with colitis, whether secreted by microglia or other cells, or peripherally sourced, have been suggested to suppress neurogenesis through different mechanisms. For instance, Cacci et al. (2005) revealed that the co-culture of an embryonic hippocampus‐derived HiB5 cell line with LPS-activated microglia results in TNFα-mediated apoptosis suppressing neuronal development and differentiation [211]. Moreover, altered hippocampal neurogenesis is seen in vivo in mice with depleted TNF receptor (TNFR)1 and TNFR2 [212]. TNFR knockout mice showed an increased rate of neural progenitor proliferation and neurogenesis in the hippocampus [212]. This study suggests that microglial activation may suppress hippocampal neurogenesis via the release of TNF-α binding to TNFR1 on hippocampal progenitors, which is known to be related to a fas-associated protein with death domain-caspase-8/3 which induces apoptosis likely contributing to impaired generation of new neurons [213] (Fig. 2). Increased hippocampal expression of TNF-α has been noted in DNBS, TNBS, and DSS-induced colitis [129, 135,136,137, 142, 143, 204]. Importantly, in DSS-colitis, cleaved caspase-8 was found upregulated in the brain and cleaved caspase-3 was found upregulated in the hippocampus, which may suggest the action of the above pathway [135, 138].

Cytokine or antigen challenge can induce microglia to release IL-1β, which has been implicated in the modulation of neurogenesis. Studies have shown IL-1R1 expression in vitro in rat embryonic forebrain NPCs [214] and adult rat hippocampal cells [215]. The binding of IL-1β is associated with decreased proliferation in hippocampal progenitor cells [216]. Furthermore, mice with chronic stress-induced depression display increased IL‐1β expression in the dorsal hippocampus that decreases dentate gyrus hippocampal neurogenesis [217]. Moreover, IL-1β dysregulation dampens BDNF secretion associated with neurodegeneration [218]. Reduced BDNF mRNA expression in the dentate gyrus and CA3 region of the hippocampus was seen in mice exposed to contextual fear conditioning followed by social isolation [218]. In vivo treatment of contextual fear-conditioned mice with an IL-1R antagonist, suppressed IL-1β signalling improving BDNF expression and preventing impairments in hippocampally-dependent contextual fear conditioning tests following social isolation [218] (Fig. 2). Limited studies in models of chemically-induced colitis provide evidence that BDNF expression is reduced in the hippocampus (DNBS) and forebrain (DSS) with IL-1β expression elevated in DSS models [135, 136, 138].

Alteration in hippocampal neurogenesis in IBD animal models may be due to abnormal excitatory synaptic properties in the hippocampus. Hippocampal tissue from Sprague–Dawley rats with TNBS-induced colitis revealed enhanced Schaffer collateral-induced excitatory field potentials in CA1 stratum radiatum [142]. Schaffer collaterals are axon collaterals from CA3 pyramidal cells projecting to CA1 area [219]. This was associated with larger-amplitude miniature excitatory postsynaptic currents (mEPSCs), but unchanged mEPSC frequencies and paired-pulse ratios, suggesting altered postsynaptic effects. Both α-amino-3-hydroxy-5-methyl-4-isoxazole propionic acid receptor (AMPA)- and N-methyl-d-aspartate (NMDA)-mediated synaptic currents were enhanced in the rats [142]. Moreover, AMPA-mediated currents revealed increased contribution of GluR2-lacking receptors and mRNA and protein levels of the glutamate ionotropic receptor AMPA type subunit 2 (GluR2) subunit were reduced in the hippocampus [142]. Interestingly, the chronic administration of minocycline, a microglial/macrophage activation inhibitor, lowered the level of TNF-α in the hippocampus and completely abolished the effect of peripheral inflammation on observed transient electrical signals and synaptic plasticity [142]. The authors had previously shown in vivo that enhanced brain excitability during colitis requires both elevated cytokines TNF-α and microglial activation [143]. Indeed, TNF-α has been evidenced to facilitate the insertion of GluR2-lacking AMPA receptors in the membrane [204, 220, 221].

The increase in hippocampal NMDA and AMPA receptors may make neurons more prone to glutamate-induced excitotoxicity, though no evidence of increased release or availability of glutamate was found [142]. The presence or absence of the GluR2 subunits determines the Ca^2+^ permeability of the AMPA receptor [222]. The low expression of GluR2 enables the formation of AMPA receptors with high Ca^2+^ permeability, which contributes to neuronal degeneration [222,223,224]. In relation to NMDA receptors, the 2A and 2B subtypes are widely distributed in the hippocampus. Moreover, quinolinate phosphoribosyltransferase, which converts NMDA agonist quinolinic acid (QA) into nicotinamide adenine dinucleotide, is low in the hippocampus reducing the capacity to clear QA in the hippocampus [225]. QA may then function as an excitotoxin and damage the hippocampal neurons [207].

It has been revealed that activation of adenosine monophosphate-activated protein kinase (AMPK) can enhance hippocampal neurogenesis through the AMPK/BDNF pathway [226]. Furthermore, there is evidence indicating that activation of AMPK attenuates inflammation in the CNS [227]. Neuroinflammation and suppression of hippocampal neurogenesis in models of colitis could be due to impairments in the AMPK/BDNF signalling pathway. A study tested this theory using an activator of AMPK, called liver hydrolysate (LH), that has been shown to increase hippocampal neurogenesis through the AMPK/BDNF pathway and has an antidepressant effect in an animal model of depression [228]. In a study using DSS-treated mice, LH prevented depressive-like behaviours and enhanced hippocampal neurogenesis through the AMPK/BDNF pathway and hippocampal activation of microglia and astrocytes [229].

HMGB1 expression could play a critical role in synaptic dysfunction and/or impaired neurogenesis in colitis models. HMGB1 is a 215 residue protein that consists of two consecutive L-shaped basic domains referred to as HMG boxes and a 30 amino-acid long tail with acidic properties [230]. HMGB1 is commonly found in the nucleus where it binds to the minor group of B type DNA and distorts and bends the double helix DNA of 90 degrees or more. HMGB1 can function to modulate transcriptional activity through its interaction with transcription factors such as p53 and nuclear factor kappa-light-chain-enhancer of activated B cells (NF-κB) [230]. Moreover, HMGB1 can function as a damage-associated molecular pattern (endogenous danger molecule released from damaged or dying cells inducing immune response by interacting with pattern recognition receptors) and bind to hippocampal TLR-4 inducing the activation of NF-κB and Activator protein 1, which facilitate the synthesis of pro-inflammatory mediators such as IL-6, TNF-α and iNOS [231]. As mentioned above, the induction of pro-inflammatory cytokines, including IL-1β, TNF-α, and IL-6, have been shown to inhibit hippocampal neurogenesis [210,211,212, 216, 217]. Using an experimental model of chronic cerebral hypoperfusion in rats, anti-HMGB1 neutralizing Ab reduced hippocampal glial activity and inflammatory cytokines, such as TNF-α, IL-1β, *and* IL-6, as well as increased antioxidants superoxide dismutase and catalase, which was associated with improved CA1 neuronal survival and cognitive tasks [232].

In DNBS-induced colitis, increased expression of the *HMGB1* gene in the hippocampus has been suggested to be detrimental to hippocampal neurogenesis and function [136]. Additionally, HMGB1 has also been shown to be involved in hippocampal neurobiological functions including memory and long-term potentiation [233, 234]. HMGB1 can inhibit hippocampal long-term potentiation and memory via TLR-4 and RAGE, which is accompanied by activation of NF-κB and c-Jun N-terminal kinase [233].

Overall, pro-inflammatory cytokines, HMGB1, glial cells and, synaptic dysfunction could independently or in unison be responsible for alterations in neurobiological pathways which promote suppression of neurogenesis seen in colitis [140, 210]. However, the exact mechanisms whereby colitis can alter pathways and the relationship between contributing factors is yet to be determined.

#### Microglia and the serotonin–kynurenine pathway

Impairments in serotonin biosynthesis could be an underlying mechanism behind behavioural changes seen in animals with colitis. However, the serotonin biosynthesis theory of depression is still debated. In treatment for tuberculosis and schizophrenia, iproniazid (inhibits the breakdown of monoamines) and imipramine (blocks serotonin and norepinephrine transport) were found to reduce depressive symptoms [235]. Moreover, reserpine, which can deplete monoamines, was implicated in triggering depressive symptoms. These observations helped formulate the theory that depression is caused by the depletion of monoamine transmission [235, 236]. But whilst serotonin biosynthesis has been implicated in depression pathogenesis, many studies have found evidence contradicting this theory. For instance, selective serotonin reuptake inhibitors increase extracellular serotonin within short periods following administration [237, 238], however, the beneficial antidepressant effects arise following weeks of continuous treatment [239]. Moreover, reducing serotonin synthesis through dietary reductions in tryptophan fails to induce depression in non-depressed individuals [240]. This review discusses the microglia-associated reduction of serotonin bioavailability as a possible mechanism underlying IBD-associated depression, however, caution should be taken as this proposed theory is still debated.

Microglial cell activity can modulate the serotonin-kynurenine pathway which plays an important role in depression [241]. Microglia express the tryptophan-catabolizing enzyme IDO in the presence of pro-inflammatory cytokines [242]. IDO converts tryptophan (amino precursor of serotonin [5-HT, 5-hydroxytryptamine]), into kynurenine (KYN) which can then be catabolized by the enzyme kynurenine 3‐monooxygenase (KMO) into excitotoxic metabolites 3‐hydroxy‐kynurenine (3‐HK), 3‐hydroxy‐anthralinic acid, QA, and finally the end‐point co‐enzyme nicotinamide adenine dinucleotide [243]. Conversely, KYN can also be metabolized through a neuroprotective pathway to kynurenic acid (KYNA) by the enzyme kynurenine–aminotransferase (KAT) [243]. Importantly, KMO is expressed by leukocytes such as monocytes, macrophages, and microglial cells, whereas KAT is present in astrocytes [242, 244]. Quinolinic acid reduces the expression of astrocyte glutamate reuptake pumps while stimulating release of glutamate from astrocytes which may result in glutamate neurotoxicity and neurodegeneration [245, 246] (Fig. 2).

Studies linking colitis with the TRY/KYN alteration in the CNS are limited. However, mice infected with a parasite *Trichuris muris (T. muris),* which induces colitis, have higher levels of serum kynurenine and an increased kynurenine/tryptophan ratio when compared to non-infected mice [247]. Moreover, *T. muris*-infected mice displayed behavioural abnormalities as assessed by a light/dark preference test and elevated levels of circulating pro-inflammatory cytokines such as TNF-α and IFN-γ which were all alleviated with either a corticosteroid (budesonide) or an anti-TNF-α agent (etanercept) interventions that normalized circulating kynurenine levels [247]. Similarly, mice with DSS and TNBS colitis revealed a reduction in serum levels of tryptophan and increased intestinal expression of IDO [248, 249]. In humans, serum obtained from CD and control participants elucidated a marked reduction in tryptophan and an increased K/T ratio in active CD. Whether these findings are due to upregulation in microglial IDO is unknown. Indeed, increased IDO-1 gene expression was observed in the medial prefrontal cortex (PFC) of mice with colitis, however, this was accompanied by reduced microglia expression [250]. It should be considered that alterations noted in serum tryptophan and K/T ratios could be due to changes in IDO-1 expression in the gut. IDO overexpressed has been noted in lesional biopsies from patients with IBD with CD123+ dendritic cells being the primary cell to express the enzyme [251]. Moreover, although appearing detrimental in the context of depression, IDO expression appears beneficial in suppressing intestinal inflammation. In TNBS colitis, inhibition of IDO results in more severe colitis and a significantly increased colonic pro-inflammatory cytokine expression [249]. This may be due to enhanced availability of tryptophan and increased 5-HT synthesis in the intestines. Indeed, 5- HT has been implicated in worsening colitis as mice with tryptophan hydroxylase-1 knockout experienced reduced 5-HT in the GI tract and had reduced severity of DSS-induced colitis [252]. It appears that there may be paradoxical findings in the brain and gut whereby reduced serotonin worsens depressive symptoms and increased serotonin contributing to more severe colitis. Overall, more research is warranted to elucidate the presence of IDO-expressing neuroglia cells in the brains of animals with colitis and whether serum levels of tryptophan and K/T ratio alteration noted in IBD and animals with colitis contribute to/or are caused by IDO expression in the CNS.

### HPA axis dysregulation in IBD

The HPA functions to coordinate neural, endocrine and immune responses to diverse stimuli that threaten physiological homeostasis. Glucocorticoids, corticosteroid hormones are the final products of HPA axis activation and function to alter cellular metabolism and the immune system [253]. The homeostatic regulation of the HPA involves bi-directional communication and integration between the brain, endocrine and immune systems [254]. The functional balance between pro- and anti-inflammatory mediators is critical for control of the HPA axis and the dysregulation in its activity, a characteristic of numerous chronic inflammatory diseases [254].

Following the immunological or emotional challenge, the hypophysiotropic neurons in the medial paraventricular nucleus (PVN) can synthesise and secrete the corticotrophin-releasing factor (CRF) into the hypophysial-portal circulation [255, 256]. CRF can then access CRF-Receptor-1 at the anterior pituitary corticotropes and stimulate the rapid release of adrenocorticotropic hormone (ACTH) [257]. ACTH enters systematic circulation and binds to melanocortin type 2 receptor in parenchymal cells of the adrenocortical zona fasciculata, which induces the release of glucocorticoids, including cortisol in humans and corticosterone in rodents [257, 258].

Inflammatory mediators, which are abnormally elevated in the serum of IBD patients [114, 115] and the brains of animals with colitis [126, 135,136,137,138,139,140,141,142,143], can also interact with the HPA axis at various points. These include (i) stimulation of vagal nerve afferents [259]; (ii) interaction with brain ECs, which induce the synthesis/release of secondary messengers such as prostaglandins [260, 261]; (iii) crossing the BBB at “leaky” regions such as the fenestrated endothelium circumventricular organs or areas where BBB dysfunction is present, whereby they activate neurons that project to the hypothalamus [262]. Inflammatory cytokines can also act directly on glucocorticoid receptors (GR) and suppress their function. For instance, activation of mitogen-activated protein kinase pathways such as ERK, JNK, and p38 by inflammatory cytokines can inhibit GR function by either directly phosphorylating GR at serine-246, indirectly via a GR co-factor, or by inhibiting translocation of the GR from the cytoplasm to the nucleus [263,264,265]. Further, cytokines can activate NF-κB, which is implicated in the inhibition of GR by the physical association in the nucleus [266].

Modulation of the HPA axis by circulating cytokines may explain evidence of a dysfunctional HPA axis in IBD patients. The cytokine IL-6, which induces cortisol secretion [267], shows no relationship to serum concentrations of cortisol in IBD patients [268, 269]. Impaired regulation of IL-6 plays a crucial part in the uncontrolled intestinal inflammatory processes in IBD. Increased formation of IL-6-sIL-6R complexes with gp130 on the membrane of CD4+ T-cells causes a STAT3-mediated transcription of anti-apoptotic genes, such as *Bcl-xl* resulting in T-cell expansion contributing to the perpetuation of chronic intestinal inflammation [270,271,272]. It has been suggested that the ANS and HPA axis are uncoupled in IBD patients as high morning vagal tone is associated with a low evening cortisol level in healthy subjects but no association was found in IBD patients [273]. Additionally, plasma NPY, a marker of ANS activity, was not positively correlated with serum cortisol in IBD patients as was observed in healthy controls [274]. In vivo*,* mice with DSS-induced colitis presented with brain region-specific alterations in HPA axis-related peptides, glucocorticoid receptor gene expression, and factors including NPY, NPY receptor Y1, CRF, CRF receptor 1, and BDNF [275, 276]. Interestingly, chemical stimulation with glutamic acid of the PVN, which is a major source of brain CRH, alleviated TNBS-evoked colitis as assessed by a reduction in colonic damage scores and levels of IL-6 and IL-17 [277]. A possible mechanism underlying these findings could be neuroinflammatory damage in brain regions implicated in the regulation of the HPA [278, 279]. Direct inflammatory insult regions of the brain could be a potential mechanism underlying HPA axis dysfunction in IBD and is supported by studies implicating neuroinflammation in animals with colitis.

A major brain region of the ANS and the stress response is the locus coeruleus (LC), which is a cluster of NA-containing neurons located in the upper dorsolateral pontine tegmentum of the brainstem [280]. Nerve fibres from the LC provide the sole source of NA to the cortex, hippocampus, cerebellum, and thalamus [280]. CRF-releasing neuronal afferent originating from the PVN project to the LC and noradrenergic neurons from the LC project to the PVN [281,282,283]. A positive feedback loop appears to exist between the HPA axis and the ANS with the firing rate of LC neurons increased by CRF which induces the release of NA by LC neurons [284,285,286]. Consequently, NA has then been shown to promote CRF mRNA expression in the PVN [287]. Consistent with this, lesions of the LC attenuate the HPA axis response to acute restraint stress but not chronic stress [288]. Importantly, it has been suggested that the Central Autonomic Network (CAN), which includes the PFC and limbic structures such as the hippocampus can exert tonic inhibitory control of the ANS, HPA axis, and amygdala [289,290,291,292].

#### The central autonomic network and neuroinflammation

**Fig. 3:**
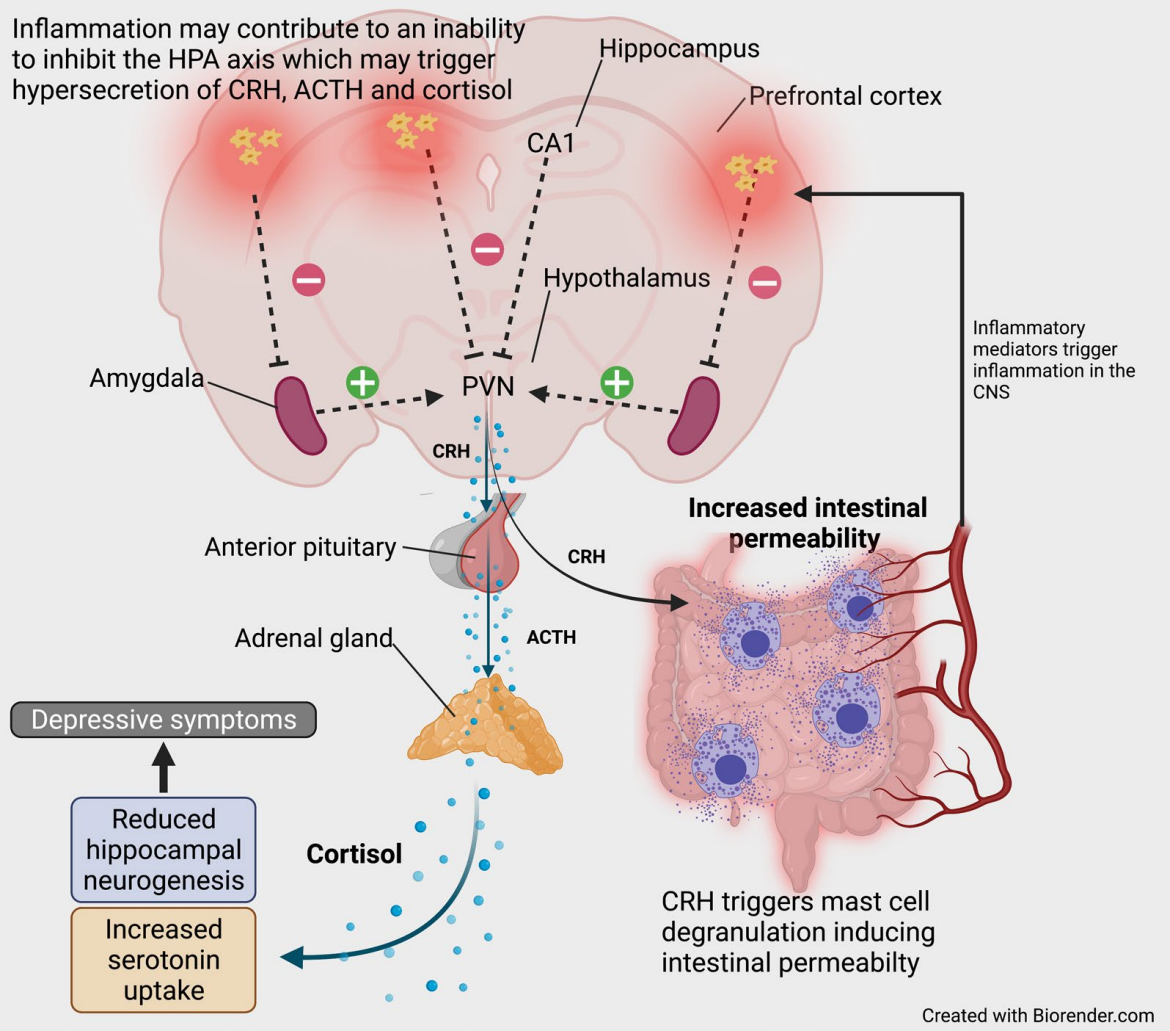
Schematic representation of neuroinflammatory-induced impairment of the HPA axis functioning. Resultant neuroinflammation associated with animal models of colitis may induce damage or neurobiological alteration in important regions associated with inhibition of the HPA axis. This may trigger the aberrant secretion of hippocampal, pituitary, or adrenal hormones triggering worsening of intestinal and cortical inflammation and depressive symptoms. *ACTH* adrenal corticotrophin-releasing hormone; *CA1* cornu ammonis 1; *CNS*, central nervous system; *CRH* corticotrophin releasing hormone; *HPA* hypothalamic–pituitary–adrenal; *PVN* paraventricular nucleus

The activity level of the ANS and HPA axis, represented by peripheral measures, such as heart rate and cortisol variability, is associated with the activity of the PFC and amygdala, respectively [293]. The amygdala is under tonic inhibition from gamma-aminobutyric acid (GABA)ergic fibres projecting from the PFC [293]. The medial and central amygdaloid nuclei are believed to stimulate the HPA axis [294]. Hence, the uncoupling between the ANS and HPA axis observed in IBD patients may be due to inflammatory insult in the PFC, as seen in animal models of IBD, which consequently impairs its role in tonic inhibition of the LC, amygdala, and HPA axis (Fig. 3). However, whether neuroinflammation or other factors contribute to proposed PFC hypoactivity in IBD patients has yet to be elucidated. Indeed, inflammation in the PFC may produce damage with varying implications on glucocorticoid release depending on regions of the PFC impacted. For instance, lesions of the prelimbic divisions (PLD) and anterior cingulate (AC) of the mPFC enhance adrenocorticotropic hormone and corticosterone secretion and induce PVN activity as determined by the neuronal activity marker c-fos following restraint stress [295,296,297]. However, lesions of the right infralimbic cortex reduce corticosterone release caused by restraint stress [298]. Importantly, the PFC is suggested to be involved in the negative feedback system, which inhibits the HPA axis. GR density is high layers II, III, and VI of the PFC [299]. In support of this function, the release of ACTH and corticosterone following restraint stress was found attenuated after infusion of glucocorticoids into the mPFC [257, 297, 300].

Studies investigating neuroinflammation in the CNS of animals with colitis should explore regions such as the PLD and AC given their suggested role in the inhibition of the HPA axis.

Studies have implicated the hippocampus in inhibition of the HPA axis [301, 302], supported by findings that the stimulation of the hippocampus decreases glucocorticoid secretion in rats and humans [303, 304]. This may be relevant to HPA axis dysfunction in IBD given that animal models of colitis provide evidence of reduced hippocampal neurogenesis, enhanced oxidative stress, mitochondrial dysfunction, and impaired synaptic transmission [136, 140, 142, 143]. Indeed, damage to the hippocampus appears to trigger a disrupted HPA response. For instance, hippocampectomy, fimbria-fornix lesions, or excitotoxic lesions increase corticosterone and/or CRH secretion [305,306,307,308]. It is believed that the CA3, dentate gyrus (including CA4), and subiculum region of the hippocampus are involved in the inhibition of the HPA axis [309], whereas the dorsal hippocampus appears to excite the HPA axis [310]. Future studies should investigate the role of these brain regions in HPA axis dysfunction evidenced in IBD patients and colitis models and ascertain whether neuroinflammatory events precipitate this dysfunction. While it could be proposed that inflammation in key regions of the brain is responsible for HPA axis dysfunction, other factors such as stress could be involved.

#### Stress and IBD

IBD patients have been shown to have a high incidence of psychological distress and comorbidities, such as depression, anxiety disorders, obsessive–compulsive disorders, and bipolar disorder [311, 312]. Stress has also been suggested to increase the susceptibility of individuals to IBD. Approximately two-thirds of patients who had both anxiety disorder and IBD developed psychiatric symptoms predating the IBD diagnosis by over 2 years with the onset of IBD also arising much earlier in patients with lifelong anxiety [313]. Recurrence and aggravation of IBD symptoms have also been shown following stressors with high perceived stress suggested as impacting the frequency of symptomatic flares [314].

Stress likely plays a role in IBD generation, recurrence, or aggravation due to impact on CRF. Stress directly influences the PVN to release CRF which can bind to CRF receptors expressed in the brain and the gut [257, 315]. Ligation of gut CRF receptors with CRF can modulate intestinal secretion, peristalsis, and the mucosal barrier permeability [316]. Moreover, CRF can trigger degranulation of mast cells that leads to enhanced mucosal permeability and promotion of pathophysiological mechanism underlying IBD [317]. Consistent with this, chronic stress in WT rats can induce intestinal barrier dysfunction, inflammatory cell infiltration, and mast cell proliferation and activation [318]. However, intestinal dysfunction in chronically-stressed mice has been abrogated in mast cell-deficient rats [318]. Further, using a mouse model of chronic restraint stress, the neuropeptide substance P and its receptors increased CRH expression and CRH release by eosinophils that resulted in a mast cell-mediated increase in epithelial barrier dysfunction [319].

Overall, whether arising due to colitis-induced central neuroinflammation, elevations of serum inflammatory mediators, or the neuroendocrinology of the stress response, inappropriate glucocorticoid secretion could have various implications in the brain and gut inflammation.

#### Glucocorticoids: good or bad in IBD?

Glucocorticoids are known for their mainly immunosuppressive action. Glucocorticoids induce the synthesis of anti-inflammatory proteins such as IL-10, lipocortin 1, and IL-1 receptor antagonists, and promote apoptosis in inflammatory cell types such as T cells and eosinophils [320, 321]. Moreover, glucocorticoids can inhibit the transcription factors AP-1 and NF-kB reducing the synthesis of several pro-inflammatory cytokines and chemokines including IL-1, IL-6, and TNF-α [322]. Treatment of severe exasperations of intestinal inflammation in IBD often involves corticosteroids, such as prednisone, hydrocortisone, and dexamethasone (DXM), to induce remission [323].

However, glucocorticoid hormones may increase intestinal inflammation and potentially worsen neuroinflammation. For instance, glucocorticoids induce the synthesis of NLRP3 inflammasome mRNA and protein and enhance ATP-mediated release of pro-inflammatory cytokines such as IL-1β, TNF-α, and IL-6 [324]. Moreover, glucocorticoids induce expression of TLR and secretion of inflammatory mediators by endothelial cells via P2Y2 purinergic receptors [325, 326]. They also modulate significant neurobiological mechanisms correlated with depression. Foetal hippocampal progenitor cells treated with corticosterone display reduced proliferation and differentiation, whereas DXM treatment only suppresses proliferation [327] (Fig. 3). Furthermore, mice treated with DXM demonstrated a cyclin-dependent kinase 5 phosphorylation of an axonal transport protein huntingtin, preventing cortical delivery of BDNF to the hippocampus resulting in impaired neurogenesis [328] (Fig. 3). Cortisol activates tryptophan 2,3-dioxygenase (TDO) which, like IDO, converts tryptophan into kynurenine [329]. These findings become even more relevant when one notes that cortisol is associated with enhanced serotonin reuptake and reductions in serotonin levels as seen in animals and humans [330, 331] (Fig. 3). Stress-induced cortisol increases intestinal barrier dysfunction, as shown by crypt analyses sourced from both humans and rodents [332]. Moreover, administration of cortisol in a porcine model caused a shift in microbiota composition [333]. These studies suggest a role of cortisol in regulating both intestinal inflammation and microbiota composition.

Through inflammation-associated damage to CNS regions responsible for its inhibition or through hypersecretion of cortisol triggered by circulating immune factors, the HPA plays a significant role in intestinal inflammation, neuroinflammation and depression in IBD patients. Future studies should ask whether regions and neural pathways of the brain responsible for inhibition of the HPA axis are damaged or fail to function appropriately in animal models of IBD. Moreover, it would be interesting to ascertain whether there is evidence of GR downregulation due to hypersecretion of cortisol in these animals.

### Dysfunction of vagal nerve tone in IBD

The vagus nerve (VN), or cranial nerve X, is a mixed parasympathetic nerve with 10–20% consisting of vagal efferent fibres and 80–90% afferent nerve fibres [334]. In the context of the GI tract, the VN’s efferent fibres transmit information to the CNS about the mechanical distortion of the mucosa, luminal osmolarity, carbohydrate levels, bacterial products, neurotransmitters, the transformation of secondary bile acids, short-chain fatty acids, branched-chain amino acids, and gut hormones [334, 335]. Sensory information travels to the nucleus tractus solitarius, located in the medulla oblongata [334, 335]. From there, nerve fibres project sensory information to different brain regions, including the ventrolateral medulla, amygdala, LC, thalamus, and hypothalamus [336]. Activation of the vagus afferent transmission can trigger the synthesis and release of the CRF by the PVN into the hypophysial-portal circulation, which then through downstream pathways discussed above, induce the release of glucocorticoids [334, 335].

Preganglionic neurons of vagal efferent fibres exit the brain from the medulla oblongata in the groove between the olive and the inferior cerebellar peduncle and in relation to the gut, innervate the muscular and mucosal layers of the gut both in the lamina propria and in the muscularis externa [334, 335]. Vagal afferent innervation of the intestines regulates the contraction of smooth muscles and glandular secretion [334, 335].

In IBD, VN tone dysfunction is suggested by vagus nerve stimulation (VNS) and functional studies. For instance, VN function was evaluated by a non-invasive test based on the heart reactions to deep breathing (*E*/*I* ratio) and tilt (acceleration and brake index). UC patients had a significantly lower *E*/*I* ratio than controls, indicating vagal nerve dysfunction [337, 338]. Furthermore, VNS significantly improved the multivariate index of colitis in rats with TNBS-induced colitis [339] and chronic VNS in the same model improved colitis and decreased the production of pro-inflammatory cytokines (TNF-α and IL-6) [340]. In patients with CD, 12-month administration of VNS restored a homeostatic vagal tone and reduced the inflammatory state [341].

Studies have suggested that both the PFC and hippocampus hold modulatory roles of VN function [342, 343]. The PFC plays an indirect role in VN tone through its role in the regulation of anatomical centres involved in emotional and stress responses, such as the amygdala [293, 344]. Similarly, studies have indicated that the hippocampus is implicated in vagal functioning [345,346,347]. Electrical stimulation of the anterior hippocampus triggers depression of cardiovascular activation with cardiovascular responses requiring an intact PFC [345] Moreover, the anterior hippocampus has connections with anatomical areas regulating stress and emotions, including medial PFC, the amygdala and various subnuclei of the hypothalamus, including the anterior hypothalamus and lateral hypothalamus [346,347,348].

The VN has been established to suppress intestinal inflammation via the cholinergic anti-inflammatory pathway [279, 349]. This is believed to be mediated by the neurotransmitter‐gated superfamily of ion channels, called α7nAChR, on macrophages [278, 279]. Stimulation of macrophage α7nAChR results in the inhibition of LPS‐mediated activation of the NF‐κB [350]. In macrophages, this effect is facilitated through the phosphorylation of Janus kinase 2 followed by activation of a STAT3 signalling pathway [278]. This causes direct inhibition of inflammatory cytokine production [278].

Upon activation, the VN releases ACh in the celiac mesenteric ganglia, which activates postsynaptic α7nAChR on adrenergic neurons of the splenic nerve, leading to the release of noradrenaline (NA) in the spleen [351]. Adrenergic nerve fibres stimulate splenic memory T cells to synthesise ACh which can interact with α7nAChR on adjacent macrophages [351]. In the intestines, the VN does not innervate directly resident macrophages but indirectly through nNOS-VIP-ACh interneurons projecting nerve endings in close proximity to resident macrophages and releasing ACh following appropriate stimulation [352].

Perhaps, neuroinflammation in brain areas implicated in the modulation of vagal tone is a contributing factor to VN dysfunction in IBD patients. Conversely, emotional and stress responses to the burden of IBD, genetics, or other unknown mechanisms may be at play. Future studies should investigate whether VN dysfunction seen in IBD patients may be triggered by neuroinflammation.

## Proposed treatment strategies

Microglial activation and production of inflammatory mediators are believed to play pivotal roles in depression and anxiety noted in models of colitis [126, 135, 136, 140, 141, 247]. Microglia or the inflammatory mediators implicated in their activation may be therapeutic targets for treating depressive symptoms as well as intestinal inflammation in IBD patients. The microglial inhibitor minocycline, a tetracycline antibiotic, suppressed LPS-stimulated inflammatory cytokine secretion and TLR expression as well as facilitating recovery from depressive behaviour and anhedonia in mice [353]. These findings were paralleled by a reduction in mRNA levels of IL-1β, IL-6, and IDO in the hippocampus and cortex, which suggests improved neuronal functioning and prevention of neurodegeneration [353]. Importantly, treatment with minocycline inhibits IFN-α induced impairment of hippocampal neurogenesis by suppressing microglial activation [354]. Minocycline is believed to selectively suppress microglial M1 polarization by inhibiting transcription and nuclear translocation of NF-κB [355]. Minocycline treatment has also shown success in treating intestinal inflammation, it reduces macroscopic and microscopic damage in intestinal tissues of TNBS-treated mice [356]. However, to date, no research has explored the neuroinflammatory and behavioural impact of microglial inhibitors in animals with colitis, which may provide valuable insight into the role of microglia in depressive symptoms in IBD patients. As pro-inflammatory cytokines are capable of reducing serotonin bioavailability and hippocampal neurogenesis, targeting these cytokines may be therapeutically beneficial in the treatment of IBD-associated psychological impairments [211, 243]. TNF-α inhibitor, infliximab, significantly improved the disease state as well as psychological functioning in IBD patients [357]. However, TNF-α inhibitors are known to cause significant immunosuppression and may increase susceptibility to infections which may outweigh any benefits as an anti-depressive treatment in these patients [358].

Antioxidants may offer a therapeutic advantage as ROS appear to be elevated in mice with colitis [135, 137]. The antioxidant, salvianolic acid B (SalB), is shown to significantly affect microglia and expression of pro-inflammatory cytokines in the cortex and hippocampus [359, 360]. In mice exposed to chronic mild stress, SalB treatment did not induce morphological changes or expression levels of microglia in the hippocampus or cortex, but induced switching from microglial M1 polarization to M2 in the hippocampus and cortex [359]. Moreover, SalB aided in the recovery of impaired neurogenesis and volumetric decreases in the dentate gyrus and the granule cell layer [359]. Additionally, rats treated with SalB showed reduced NLRP3 inflammasome formation in the CA1 region of the hippocampus and restored autophagy function following the LPS challenge [360]. This suggests that SalB may promote autophagy clearance of excessive NLRP3 formation, suppressing the formation of NLRP3 pro-inflammatory cytokines such as IL-18 and IL-1β [360]. Within the intestines, SalB has been shown to improve intestinal barrier tight junction dysfunction in mice with IL-1β-induced colitis [361].

Another promising therapeutic could be hydrogen-rich water (HRW), a potent antioxidant, which can penetrate the cell membrane and selectively reduce hydroxide radicals and peroxynitrites without influencing physiological ROS [362, 363]. This has shown promising results in managing intestinal inflammation. Mice with DSS-induced colitis treated intraperitoneally with HRW showed reduced disease severity, pro-inflammatory cytokine production, and oxidative stress markers compared to untreated mice with DSS-induced colitis [364]. Importantly, mice given HRW and exposed to chronic unpredictable mild stress (CUMS) exhibit reduced ROS expression in the hippocampus and prefrontal cortex compared to mice exposed to CUMS without HRW [365]. Moreover, the HRW-treated group had significant reductions in IL-1β and inflammasome enzyme caspase-1 in the hippocampus and cortex, and did not experience depressive symptoms compared to the untreated group following CUMS [365].

Given there has been evidence of reduced antioxidant GSH in mice with colitis, administration of GSH may offer therapeutic benefits for depressive symptoms [136]. This is assumed given that reduction in GSH has been noted in post-mortem PFC samples of patients with various psychiatric diseases such as MDD, schizophrenia, and bipolar disorder [147]. There appear to be limited studies exploring the therapeutic benefits of direct GSH treatment, however, the antioxidant precursor to GSH, N-acetylcysteine, which can raise brain GSH levels, has shown some therapeutic success in treating depression [366]. GSH may also aid in treating intestinal inflammation as IBD mucosal samples show the deficiency of GSH and hypoactivity of the enzyme producing GSH, γ-glutamyl cysteine synthetase [367].

Expression of NOS in the hippocampus was paralleled with depressive symptoms observed in mice with colitis [136, 137]. Moreover, excessive production of NO by iNOS is noted in the inflamed gut of patients with IBD; inhibition of iNOS reduced the severity of intestinal inflammation in animal models of colitis [368, 369]. Therefore, targeting NOS with inhibitors may offer treatment for intestinal inflammation and depression. Promising results have been seen in mice with TNBS-induced colitis, in which administration of the NOS inhibitor N-nitroarginine methyl ester resulted in an anti-depressant effect determined by reduced immobility time in the forced swim test [137].

Whilst no studies have shown COX upregulation in the brains of animals with colitis, it is known that inflammatory signalling can induce COX1 and 2 expression by microglia in rodent and human brains and correlates with neurodegenerative changes [370]. Hence, if they are present in the brains of IBD models, targeting COX enzymes may be a novel approach for the treatment of depression. This is premised on studies where patients receiving the COX-2 inhibitor, celecoxib, with the antidepressant sertraline or reboxetine had a greater improvement in depressive symptoms compared to the sertraline/reboxetine only group [371, 372].

## Conclusions

This review aimed to describe the structural and physiological alterations in the GI tract, blood circulation, and the CNS in IBD patients and corresponding animal models of IBD in detail. The findings support the idea that CNS neuroinflammation is either a cause or contributor to the depression so often seen in IBD patients. Moreover, potential new neurobiological or intestinal targets for future studies have been revealed for the development of better therapeutic options for IBD-associated depression. Importantly, many underlying mechanisms of depression comorbid with IBD may be highly translatable to other systemic inflammatory diseases such as rheumatoid arthritis, obstructive pulmonary disease, and diabetes, which exhibit higher rates of depression compared to the general population.

## Data Availability

Not applicable.

## Abbreviations

5-HT: 5-Hydroxytrptamine
Aβ: Amyloid-β peptide
AC: Anterior cingulate
Ach: Acetylcholine
AMPK: Adenosine monophosphate-activated protein kinase
ANS: Autonomic nervous system
AQP4: Aquaporin-4
BBB: Blood brain barrier
BCEC: Brain cerebral endothelial cells
BDNF: Brain-derived neurotrophic factor
CA1: Cornu ammonis
Caco: Colorectal adenocarcinoma
CBR: Cannabinoid receptor
CCL: Chemokine (C–C motif) ligand 2
CD: Crohn’s disease
CNS: Central nervous system
COX: Cyclooxygenase
CRH: Corticotrophin releasing hormone
CSF: Cerebral spinal fluid
CUMS: Chronic unpredictable mild stress
CVO: Circumventricular organ
DSS: Dextran sulfate sodium
EC: Endothelial cell
EGC: Enteric glial cell
ENS: Enteric nervous system
FADD: Fas-associated protein with death domain
FITC: Fluorescein isothiocyanate
GABA: Gamma aminobutyric acid
GDNF: Glial cell line-derived neurotrophic factor
GFAP: Glial fibrillary acidic protein
GI: Gastrointestinal
GLU: Glutamate
GR: Glucocorticoid receptor
GSH: Glutathione
GSNO: Glial S-Nitrosoglutathione
HMGB1: High mobility group box protein 1
HRW: Hydrogen rich water
Iba-1: Ionized calcium-binding adapter molecule 1
IBD: Inflammatory bowel disease
IDO: Indoleamine-pyrrole 2,3-dioxygenase
IFN-γ: Interferon gamma
Ig: Immunoglobulin
IL: Interleukin
iNOS: Inducible nitric oxide synthase
ISF: Brain interstitial fluid
JAM: Junction adhesion molecule
KAT: Kynurenine aminotransferase
KMO: Kynurenine 3-monooxygenase
KYN: Kynurenines
KYNA: Kynurenic acid
LC: Locus coeruleus
LH: Liver hydrolysate
LPS: Lipopolysaccharide
MDD: Major depressive disorder
MLC: Myosin light chain
MLCK: Myosin light chain kinase
MMP: Metalloproteinase
mRNA: Messenger ribonucleic acid
NE: Norepinephrine
NF-kB: Nuclear factor kappa-light-chain-enhancer of activated B cells
NLRP: Nucleotide-binding oligomerization domain
NO: Nitric oxide
NOS: Nitric oxide synthase
NPY: Neuropeptide Y
P: Phosphate
PFC: Prefrontal cortex
PGE2: Prostaglandin E2
PI3K: Phosphoinositide 3-kinase
PLD: Prelimbic division
QA: Quinolinic acid
ROCK: Rho-associated protein kinase
ROS: Reactive oxygen species
SHH: Sonic hedgehog
STAT3: Signal transducer and activator of transcription 3
T. muris: Trichuris muris
TEER: Transepithelial/transendothelial electrical resistance
TGF-β: Transforming growth factor beta
Th: T-helper
TJ: Tight junction
TLR: Toll-like receptor
TNBS: Trinitrobenzenesulfonic acid
TNF-α: Tumour necrosis factor alpha
TNFR: Tumour necrosis factor receptor
TRY: Tryptophan
UC: Ulcerative colitis
VIP: Vasoactive intestinal peptide
VN: Vagus nerve
VNS: Vagal nerve stimulation
WT: Wild type
ZO: Zonula occludens
